# Age-Related Mortality in STEMI Patients: Insight from One Year of HUB Centre Experience during the Pandemic

**DOI:** 10.3390/jcdd9120432

**Published:** 2022-12-02

**Authors:** Gabriele Tumminello, Andrea D’Errico, Alessio Maruccio, Domitilla Gentile, Lucia Barbieri, Stefano Carugo

**Affiliations:** 1 Department of Cardio-Toracic-Vascular Diseases, Foundation IRCCS Ca’ Granda Ospedale Maggiore Policlinico, Via Francesco Sforza, 35, 20122 Milano, Italy; 2 Cardiovascular Research Team, San Carlo Clinic, Via Bertola, 3, 20026 Novate Milanese, Italy; 3 Department of Clinical Sciences and Community Health, University of Milan, 20122 Milan, Italy

**Keywords:** STEMI, age, COVID-19

## Abstract

Background: Old patients have a poor prognosis when affected by ST elevation myocardial infarction (STEMI). The aim of our study was to evaluate the impact of age on acute and mid-term mortality in STEMI patients over one year in the pandemic period. Methods: we collected data on 283 STEMI patients divided into three groups according to age (not old, “Not-O”, ≤74 y/o; old, “O”, 75–84 y/o; very old, “Very-O”, ≥85 y/o). Results: the three groups did not differ in their clinical or procedural characteristics. The Very-O patients had a significantly increased incidence of in-hospital MACE (35%), mortality (30.0%), and percentage of cardiac death (25.0%). The only two independent predictors of in-hospital mortality were the ejection fraction (EF) [OR:0.902 (95% CI) 0.868–0.938; *p* < 0.0001] and COVID-19 infection [OR:3.177 (95% CI) 1.212–8.331; *p* = 0.019]. At follow-up (430 +/− days), the survival rates were decreased significatively among the age groups (Not-O 2.9% vs. O 14.8% vs. Very-O 28.6%; *p* < 0.0001), and the only two independent predictors of the follow-up mortality were the EF [OR:0.935 (95% CI) 0.891–0.982; *p* = 0.007] and age [OR:1.06 (95% CI) 1.018–1.110; *p* = 0.019]. Conclusions: in very old patients, all the accessory procedures that may be performed should be accurately and independently weighed up in terms of the risk–benefit balance and the real impact on the quality of life because of the poor mid-term prognosis.

## 1. Introduction

The global number of old and very old patients is constantly increasing due to the improvements in socio-economic conditions and the quality of care. Age is known to be a major non-modifiable cardiovascular risk factor, and older age is associated with severe cardiovascular comorbidity [1]. The exact prevalence and incidence of acute coronary syndrome (ACS) in patients over 75 years of age are unknown. However, patients over 65 years of age account for 60% of all hospitalizations due to ACS. Furthermore, 85% of deaths associated with myocardial infarction occur in this patient group [2]. It is known that the incidence of ACS is decreasing in the younger population, while the proportion of elderly ACS patients is still increasing [3]. Several registries [4] show that ACS mortality is higher in the elderly population than in the younger population. In older people with a history of ST elevation, myocardial infarction (STEMI) mortality rates are increased by over 30%. The management of ACS in the elderly population is theoretically identical to that in younger patients; however, there is no shared consensus on this topic. The success rate of percutaneous coronary intervention (PCI) among elderly patients is lower than in younger patients [5], even in the subset of STEMI older patients, who are affected by a higher rate of unsuccessful reperfusions and a higher mortality with respect to young patients (8.5% vs. 2.6%, respectively) [6]. Nonetheless, older age does not represent a contraindication to primary angioplasty (pPCI), and even in older multimorbid patients, the invasive approach improves the prognosis with respect to the conservative one [7]. Beyond the acute condition, elderly patients often have more diffuse coronary artery disease, with more complex, calcified, and rapidly progressive lesions and with greater involvement of the left main coronary artery and a consequently worse prognosis [8]. During the pandemic, there has been an effective reorganization of the emergency systems, with the identification of 13 HUB centers in Lombardy for the treatment of STEMI [9]. The number of ACS patients hospitalized was lower, in particular, during the first pandemic phase, probably due to the fear, especially among elderly, of COVID-19 infection. The late diagnosis of, and co-infection with, COVID-19 contributed to a poor prognosis [10,11]. The aim of our study was to evaluate the impact of age, focusing on old and very old patients, on acute and mid-term mortality in STEMI patients hospitalized during the pandemic period. 

## 2. Materials and Methods

We retrospectively analyzed all the patients hospitalized at our HUB center from 15 March 2020 to 15 March 2021 with a diagnosis of STEMI. The study was performed in accordance with the Declaration of Helsinki, and all the patients signed a disclosure form for the use of the personal data that were collected anonymously in a dedicated database. Hypertension and diabetes mellitus were defined according to the contemporary guidelines [12,13]. Blood data were collected upon admission to hospital, except for the peak values of the cardiac biomarkers. The ejection fraction (EF) was calculated using standard transthoracic echocardiography method with Simpson’s biplane by two expert operators independently before discharge, and the final value was the median of the two results. The diagnosis of SARS-CoV infection was determined by a nasopharyngeal PCR-based swab test at the time of admission and repeated during the hospitalization period if deemed necessary. Cardiac death was defined as secondary to fatal arrhythmias, cardiogenic shock, or mechanical complications of STEMI. The characteristics of ventilatory assistance were recorded upon admission and monitored during hospitalization. Respiratory complications were defined as respiratory worsening requiring an increase in ventilatory support compared to admission. Death from all causes, ischemic stroke, non-fatal myocardial infarction, and unscheduled urgent revascularization were recorded as in-hospital adverse events (MACE). Coronary angiography was performed through both the radial and femoral approaches following the clinical indication of expert operators. Coronary artery critical stenosis was defined as a >70% stenosis of the involved vessel with a minimum diameter of ≥1.5 mm. Multivessel disease was defined as the presence of stenosis in at least two major vessels (≥2 mm). The total ischemic time (time from the onset of chest pain to the restoration of the coronary flow) was calculated for each patient. At the end of the procedure, the thrombolysis in myocardial infarction (TIMI) degree of coronary flow was detected to ensure a uniform method of assessing coronary reperfusion. The success of the procedure was defined as cases where a TIMI flow of >2 was restored in all the coronary branches. Therapy at discharge was prescribed according to the current guidelines and clinical judgment. Follow-up and related events were finally collected through direct visits, phone contact, or electronically from the “Registro Nazionale Lombardia”. Statistical analyses were performed with the SPSS 23 statistical package. Continuous variables were expressed as the mean ± standard deviation, and categorical variables were expressed as percentages. ANOVA and Chi^2^ tests were used to compare the continuous and categorical variables between the three groups. The Cox regression model and univariate and multivariate logistic analyses were used to identify the independent predictor variables associated with follow-up and in-hospital events. The cumulative long-term mortality was estimated using Kaplan–Meyer analysis and compared with the log-rank test. A *p* value of < 0.05 was considered statistically significant, and the confidence intervals (CI) were calculated at 95%. 

## 3. Results

**Figure 1:**
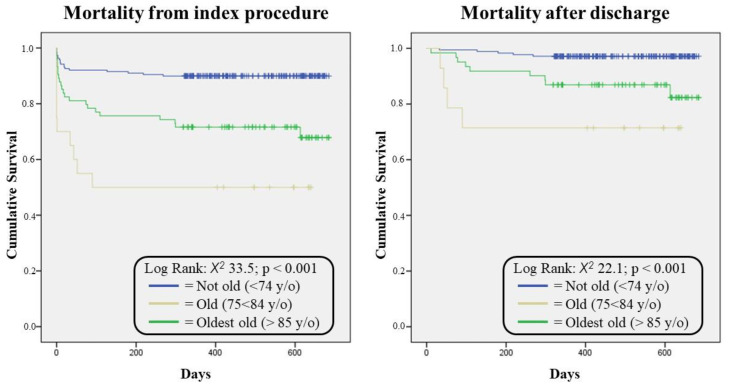
The cumulative survival rates of the three groups.

From a total of 283 STEMI patients, we divided our population into three groups according to age (not old, “Not-O”, ≤74 y/o; old, “O”, 75–84 y/o; and very old, “Very-O”, ≥85 y/o) [14]. The clinical characteristics are reported in Table 1. The procedural and therapeutical characteristics, COVID-19 characterizations, and follow-up data are listed in Table 2. In the Very-O group, cardiovascular risk factors such as hypertension, previous smoking status, and diabetes showed a greater distribution, except for dyslipidemia and overweight. Regarding the STEMI diagnosis, the O group showed a higher frequency of in-hospital diagnosis and a relative reduction in the pre-hospital setting. A high Killip class (III/IV) was recorded in 12.1% of the total population, with a higher prevalence in the Very-O group (31.6%; *p* = 0.05). Regarding the echocardiographic assessment, the EF showed significant variations between the three groups, reaching 40.1 +/− 13.0% in the Very-O compared to 49.8 +/− 9.7% in the Not-O group, with a clear, significant reduction with increasing age. The blood chemistry tests did not show large significant differences, except for the hemoglobin levels (12.7 +/− 2.0 g/dL in Very-O vs. 13.7 +/− 1.9 g/dLin the total population; *p* < 0.0001) and creatinine (1.27 +/− 0.56 mg/dL in Very-O vs. 1.00 +/− 0.34 mg/dL in the total population; *p* < 0.0001), again with a significative trend of decrease and increase, respectively, with increasing age. The low-density lipoprotein (LDL) cholesterol values were lower in the Very-O group (82 +/− 28 mg/dL; *p* = 0.01). The COVID-19 infection incidence and incidence of mechanical ventilatory support received by the patients were 8.7% and 7.2%, respectively, among the entire population, with no significant differences between the three groups. Regarding the STEMI treatment, all the patients were treated with primary PCI (pPCI), with no differences in terms of their procedural access or success rate. The patients were similar in terms of multivessel involvement. The most frequently represented MI, independent from age, was anterior STEMI (45.9%). Procedural success in the global population was achieved in 96.1% of cases, not differentiating between the three subgroups. Multivessel disease was treated with staged in-hospital complete revascularization in 62.4% of cases, with no differences between groups (Not-O 67.0% vs. O 53.4% vs. Very-O 55.1%; p:ns). Among the patients with no complete revascularization, none were scheduled for staged PCI at follow-up. The total ischemic time was 273 +/− 367 min in the whole population and homogeneously distributed between the subgroups. The discharge therapy showed a reduced prescription of ACE inhibitors (ACEi), beta-blockers, and direct oral anticoagulants (DOAC) in the over-85-year-old population compared to the other two groups. Most of the patients were treated with double antiplatelet therapy, in 90.6% of cases with a combination of acetylsalicylic acid and ticagrelor. The Very-O patients had a significantly increased incidence of MACE with respect to the other groups (Not-O 10.6% vs. O 24.3% vs. Very-O 35.0%; *p* < 0.001), as well as a higher in-hospital mortality rate (Not-O 7.4% vs. O 17.6% vs. Very-O 30.0%; *p* = 0.02) and a significant percentage of cardiac deaths (Not-O 4.2% vs. O 13.5% vs. Very-O 25.0%; *p* < 0.001). No differences in terms of respiratory complications were registered among the three subgroups. The mean hospitalization period was 9.4 +/− 7.3 days, with no differences between groups. The median follow-up in the population was 464 days, with an interquartile distance of 271 days. All-cause death at follow-up, including the hospitalization period, was 18% for the global population, with a significant increase with age (Not-O 10.1% vs. O 29.7% vs. Very-O 50.0%; *p* < 0.0001). Focusing on the deaths that occurred after discharge, the findings were similar, with a significant increase with age (Not-O 2.9% vs. O 14.8% vs. Very-O 28.6%; *p* < 0.0001). The cumulative survival rates of the three groups are shown in Figure 1, both including and excluding the in-hospital period. According to the univariate and multivariate analyses, the only two independent predictors of in-hospital mortality for the entire population were the EF [OR: 0.902 (95% CI) 0.868–0.938; *p* < 0.0001] and COVID-19 infection [OR: 3.177 (95% CI) 1.212–8.331; *p* = 0.019]. For this, see Table 2. When performing the univariate and multivariate analyses of the three age subgroups (see Table 3), the only independent predictor of in-hospital mortality that was constant across the three groups was the EF [Not-O OR: 0.893 (95% CI) 0.828–0.962; *p* = 0.003, O OR: 0.919 (95% CI) 0.876–0.965; *p* = 0.001, and Very-O OR: 0.905 (95% CI) 0.832–0.984; *p* = 0.019]. According to the Cox regression model univariate and multivariate analyses, the only two independent predictors of mid-term follow-up mortality for the entire population were the EF [OR: 0.935 (95% CI) 0.891–0.982; *p* = 0.007] and age [OR: 1.06 (95% CI) 1.018–1.110; *p* = 0.019]. Similar statistical analyses were performed separately on the COVID subgroups, but the small sample size limited all the power of the findings.

## 4. Discussion

Among old STEMI patients hospitalized during the pandemic, this study found an increased short- and mid-term mortality rate proportional to age. The treatment of very old STEMI patients is always challenging due to the complexity of the lesions and the frailty of the patients themselves. Regarding the lesion’s complexity, old patients present with more calcified and multivessel CAD, with an increased involvement of the left main artery [15,16]. Our population showed a trend of more multivessel involvement, but it was not statistically significant. Conversely, our population confirmed the frailty of this group of patients, as they presented with a higher prevalence of cardiovascular risk factors, with the exclusion of hypercholesterolemia. This finding is easily explained by the more frequent prescription of hypolipemic therapy treatment before hospitalization with respect to younger patients, for whom this had been their first cardiovascular event. Despite the patient and CAD complexity, all the subjects in our population were treated successfully and equally independent of age, with the success and complete revascularization rates being similar among the different age groups. The only discrepancy was the null use of IABP in the Very-O group, even with 10% of cases presenting with cardiogenic shock. This may underline the operator’s hesitation to apply aggressive treatments to complex and elderly patients. Nevertheless, the rate of IABP should not have influenced our findings in terms of the mortality rate, as previously described [17]. Lesion complexity and patient frailty may have influenced the final reduced EF in the oldest old group. The EF, as an expression of myocardial injury and residual pump function [18], was one of the independent predictors of in-hospital mortality. The reduced EF may explain the higher in-hospital mortality rate of the Very-O patients. Even if the other independent predictor of in-hospital mortality was COVID-19 infection, this does not account for the increased Very-O group mortality. In fact, respiratory complications and ventilatory support were similar across the three age-stratified groups. Discharge medical therapy was similar among the three groups, with a lower level of prescription in the Very-O group of beta-blockers, ACE-I, and DOAC, prescribed for the hypotensive side effect or a higher presence of high-risk bleeding in the older group. All the patients, regardless the age group, were treated equally with the invasive approach, and a high success rate was achieved, probably due to the high-volume cat-lab and the expertise of the operators. The higher mortality rate of the Very-O group with respect to the O and Not-O patients is not only related to the acute phase of the STEMI. Even if the Very-O patients were treated similarly to the other age groups, in the follow-up, the mortality rate remained elevated. The Very-O group showed a mortality rate after discharge of up to 28% within a year. The only variable independently related to the mid-term follow-up was age. The invasive treatment of very old patients is debated, but it seems to provide a benefit in regard to the mortality rate under several different conditions [19,20]. In our population, all the patients received the same treatment, with a high success rate regardless of age, but the Very-O group remained affected by a high-grade mid-term mortality. The effect of age on mortality is clearly evidenced in Figure 1, where, regardless of whether the intra-hospital period is considered or not, the mortality rate increases with the increase in age per group, especially in the first period after discharge, where the three curve curves clearly differ. The figure graphically expresses the concept that, among very old patients, even if we consider only the survivors of acute events, who are discharged and treated equally to patients who are not old, they are affected by a high mortality rate. Limitations: The main limitation is that the study is a single-center retrospective study, but it was conducted in a HUB center during the pandemic, permitting us to collect a sufficient number of STEMI patients. A second limitation is the follow-up, which we limited to the survival rate, and the individual causes of death were not registered, preventing us from distinguishing cardiovascular deaths from other causes, which may have had considerable and different impacts on the three different age groups. 

## 5. Conclusions

Our findings suggest that in a population of STEMI patients, the mortality increases with age, even if the very old are treated the same as younger patients. The relative lack of benefits of applying a standard and invasive treatment implies the need to carefully consider the risk/benefit ratio before submitting old patients to any invasive procedure, because they have, in all cases, a poor mid-term prognosis.

## Figures and Tables

**Table 1:** Clinical Characteristics.

Clinical Characteristics (*n*)	Tot (283)	Not-O ≤ 74 y/o (189)	O 75–84 y/o (74)	Very-O ≥ 85 y/o (20)	*p*
Age (years)	66.7 +/− 12.7	59.6 +/− 8.8	79.0 +/− 3.7	87.9 +/− 2.9	<0.0001
BMI (kg/m^2^)	26.4 +/− 4.3	26.8 +/− 4.2	25.2 +/− 4.2	26.7 +/− 5.4	0.02
Hypertension (%)	55.2	45.7	78.4	84.2	<0.0001
Never smoked (%)	54.4	45.7	71.6	73.7	<0.0001
Active smoker (%)	33.4	45.7	13.5	5.3	
Previous smoker (%)	10.7	8.5	14.9	21.1	
Dyslipidemia (%)	28.6	31.4	28.4	15.8	ns
Diabetes (%)	20.0	16.5	27.0	36.8	0.03
Obesity(%)	20.0	23.9	12.2	21.1	ns
F. history of CAD (%)	13.4	18.6	4.1	5.3	0.005
Previous MI (%)	13.4	12.2	20.3	5.0	ns
Previous PCI (%)	13.1	12.7	16.2	10.0	ns
Previous CABG (%)	2.8	2.6	2.7	5.0	ns
COPD (%)	9.0	5.3	13.5	31.6	<0.0001
Dialysis (%)	1.1	0.5	2.7	0	ns
**MI type**					
Anterior MI (%)	45.9	48.4	40.5	60.0	ns
Inferior MI (%)	39.7	38.8	45.9	40.0	
Lateral MI (%)	9.0	8.5	13.5	0	
Posterior MI (%)	2.8	4.3	0	0	
**Site of diagnosis of MI**					
Pre-hospital (%)	61.0	66.7	45.2	65.0	0.009
Emergency Room (%)	33.3	28.0	49.3	25.0	
*Spoke* center (%)	3.5	3.7	1.4	10.0	
*Hub* hospital Dep. (%)	2.1	1.6	4.1	0	
Out of hospital CA (%)	7.2	10.1	2.7	0	ns
Cardiogenic Shock (%)	7.9	7.4	9.5	10.0	ns
Killip Class>2 (%)	12.1	10.5	13.5	31.6	0.03
EF (%)	48.3 +/− 11.0	49.8 +/− 9.7	46. 8 +/− 12.5	40.1 +/− 13.0	<0.0001
**Biochemistry**					
Hemoglobin g/dL	13.7 +/− 1.9	14.1 +/− 1.7	13.0 +/− 2.1	12.7 +/− 2.0	<0.0001
Creatinine mg/dL	1.00 +/− 0.34	0.93 +/− 0.28	1.10 +/− 0.37	1.27 +/− 0.56	<0.0001
Glycemia mg/dL	155 +/− 72	73 +/− 5	71 +/− 8	67 +/− 15	ns
Total cholesterol mg/dL	171 +/− 46	175 +/− 43	166 +/− 56	149 +/− 22	ns
LDL cholesterol mg/dL	104 +/− 41	109 +/− 41	98 +/− 43	82 +/− 28	0.01
Troponin T ng/L	6157 +/− 3224	5902 +/− 11000	6561 +/− 10402	7085 +/− 7780	ns

All the continuous variables are expressed as mean± standard deviation. Abbreviations: F. history of CAD: familiar history of coronary artery disease; MI: myocardial infarction; PCI: percutaneous coronary intervention; CABG: coronary artery bypass graft; COPD: chronic obstructive pulmonary disease; MI: myocardial infarction; Dep.: department; CA: cardiac arrest; EF: ejection fraction.

**Table 2:** Procedural, COVID-19, and therapeutical characteristics and events.

Procedural Characteristics (*n*)	TOT (283)	Not-O ≤ 74 y/o (189)	O 75–84 y/o (74)	Very-O ≥ 85 y/o (20)	*p*
Radial approach (%)	73.8	77.1	74.3	70.0	ns
Multivessel disease (%)	54.5	53.7	58.1	70.0	ns
N° of critical vessels	1.78 +/− 0.80	1.71 +/− 0.79	1.92 +/− 0.82	1.90 +/− 0.85	ns
Critical left main (%)	5.5	3.2	12.2	5.0	0.01
Culprit vessel LAD (%)	45.0	45.2	42.5	52.6	ns
SYNTAX score	17.7	16.8	16.9	19.2	ns
IABP (%)	4.8	4.2	8.1	0	ns
Final TIMI flow ≥ 2 (%)	96.1	97.3	91.8	100	ns
Comp. revasc. during index procedure (%)	4.5	11.0	2.3	7.1	ns
Comp. revasc. during hospitalization (%)	62.4	67.0	53.5	55.1	ns
Total ischemic time (min)	273 +/− 367	262 +/− 390	302 +/− 324	269 +/− 300	ns
**COVID-19 characterization**					
COVID-19 + (%)	8.6	9.6	8.2	5.0	ns
Ventilatory mechanical support (%)	7.2	9.1	10.9	18.2	ns
**Therapy at discharge**					
B-blocker (%)	88.2	91.7	84.8	62.5	0.002
ACE-I (%)	71.0	77.2	59.1	50.0	0.003
ARBs (%)	9.2	7.2	15.2	7.3	ns
Acetylsalicylic acid (%)	98.9	99.5	97.1	100	ns
DAPT (%)	93.3	95.2	90.5	85.0	ns
Statin (%)	97.0	96.7	98.5	93.8	ns
Direct anticoagulant (%)	6.1	2.8	15.2	6.3	0.002
**In-hospital events**					
In-hospital mortality (%)	11.4	7.4	17.6	30.0	0.02
MACE (%)	15.9	10.6	24.3	35.0	0.001
Respiratory complications (%)	6.4	5.8	8.1	5.0	ns
Cardiac death (%)	7.9	4.2	13.5	25.0	0.001
Hospitalization days	9.4 +/− 7.3	8.8 +/− 7.3	10.6 +/− 7.5	11.0 +/− 4.4	ns
**Follow-up events**					
Follow-up (days)	430 +/− 208	459 +/− 178	396 +/− 238	283 +/− 276	0.0001
Follow-up median and interquartile distance (days)	464–271	467–245	458–391	247–580	
Death from any cause from index procedure (%)	18.0	10.1	29.7	50.0	0.0001
Death from any cause after discharge (%)	7.2	2.9	14.8	28.6	0.0001

All the continuous variables are expressed as mean± standard deviation. Abbreviations: LAD: left anterior descendent; IABP: intra-aortic balloon pump; Comp. revasc.: complete revascularization; ACE-I: angiotensin-converting enzyme inhibitors; ARB: angiotensin receptor blockers; DAPT: double antiplatelet therapy; MACE: major adverse cardiovascular event.

**Table 3:** Multivariate analysis of in-hospital mortality.

Variable		OR	[CI]	*p*
	**Overall Population**			
EF		0.90	0.868–0.938	<0.0001
COVID-19 +		3.17	1.212–8.331	ns
Killip class > 2		2.12	0.918–4.935	0.07
Age		1.00	0.963–1.039	ns
	**Not Old ≤ 74 y/o**			
EF		0.893	0.828–0.962	0.003
COVID-19 +		1.995	0.491–8.106	ns
Killip class > 2		1.099	0.189–6.396	Ns
Out of hospital CA (%)		4.283	0.777–23.619	0.095
	**Old 75–84 y/o**			
EF		0.919	0.876–0.965	0.001
Killip class > 2		1.622	0.431–6.107	ns
	**Very Old ≥ 85 y/o**			
EF		0.905	0.832–0.984	0.019

Abbreviations: CA: cardiac arrest; EF: ejection fraction.

## Data Availability

Not applicable.
